# Woodlice and their parasitoid flies: revision of Isopoda (Crustacea, Oniscidea) – Rhinophoridae (Insecta, Diptera) interaction and first record of a parasitized Neotropical woodlouse species

**DOI:** 10.3897/zookeys.801.26052

**Published:** 2018-12-03

**Authors:** Camila T. Wood, Silvio S. Nihei, Paula B. Araujo

**Affiliations:** 1 Federal University of Rio Grande do Sul, Zoology Department. Av. Bento Gonçalves, 9500, Prédio 43435, 91501-970, Porto Alegre, RS, Brazil Federal University of Rio Grande do Sul Porto Alegre Brazil; 2 University of São Paulo, Institute of Biosciences, Department of Zoology. Rua do Matão, Travessa 14, n.101, 05508-090, São Paulo, SP, Brazil University of São Paulo São Paulo Brazil

**Keywords:** Diptera, Isopoda, Oniscidea, parasitoids, Rhinophoridae

## Abstract

Terrestrial isopods are soil macroarthropods that have few known parasites and parasitoids. All known parasitoids are from the family Rhinophoridae (Insecta: Diptera). The present article reviews the known biology of Rhinophoridae flies and presents the first record of Rhinophoridae larvae on a Neotropical woodlouse species. We also compile and update all published interaction records. The Neotropical woodlouse *Balloniscusglaber* was parasitized by two different larval morphotypes of Rhinophoridae. Including this new record, there are 18 Isopoda species known to be parasitized and 13 Rhinophoridae species with known hosts, resulting in 35 interactions. There are a total of 53 interaction records from Holarctic and Neotropical countries. Of the 18 known isopod hosts, only five species have more than one parasitoid, including the new Neotropical host record presented in this work.

## Introduction

Terrestrial isopods are soil macroarthropods involved in decomposition processes and nutrient cycling (Zimmer 2002). This group has many predators within the soil but few known parasites and parasitoids. Among parasitoids, all known species belong to the family Rhinophoridae (Insecta: Diptera) (Sutton 1980). This family of flies comprises about 150 species worldwide that mainly parasitize woodlice (Pape and Arnaud 2001, Nihei 2016). Despite their numbers, not many papers discuss the woodlouse-parasitoid interaction. Studies regarding the interaction and fly’s larval stages are scarce and difficult to find and the taxonomy and phylogeny of both groups have been considerably modified since those studies were published. Hence, there is no current list of recorded interactions and a need to update them taxonomically. Information from immature stages and their biology is crucial for evaluating the systematic position of many aberrant oestroid flies such as the rhinophorids (Pape and Arnaud 2001), so knowledge of the morphology of larval stages may help phylogenetic analysis and classification (Cerretti et al. 2014), as well as to understand its evolutionary history in association with the woodlice hosts. Therefore, this work aims to (1) review the known biology of Rhinophoridae larvae focusing on the woodlouse-larva interaction, (2) present the first record of Rhinophoridae larvae on a Neotropical woodlouse species and (3) update the recorded interactions according to current taxonomy of both groups.

## Material and methods

Bibliographic searches in the platforms Web of Science, Science Direct, Biodiversity Heritage Library and Google Scholar were performed using the following keywords: Rhinophoridae, woodlouse flies, Tachinidae, Rhinophorinae. All the subsequent references from obtained papers were searched in available databases and scientific libraries.

Regarding the new woodlouse host record, infected individuals of *Balloniscusglaber* Araujo & Zardo, 1995 that had been collected in Morro Santana, Porto Alegre, Southern Brazil (30°4'4"S, 51°7'22"W) were discovered from laboratory culture. The location is at 100 m of elevation and the vegetation consists of a mosaic of Atlantic forest and grassland (Overbeck et al. 2006). Hosts were carefully dissected, photographed, and preserved in ethanol 70 %. Larvae were heated in water at 60 °C before being transferred to ethanol whenever possible. The material used in this study is deposited in Museu de Zoologia, Universidade de São Paulo, São Paulo, Brazil (MZUSP).

Taxonomy of isopod species was updated according to Schmalfuss (2003) and recent revisions. Taxonomy and name validity of Rhinophoridae species were based on regional catalogues and recent generic revisions, when available (Herting 1993, Cerretti and Pape 2007, 2009).

## Results and discussion

### Biology of larval stages: Isopoda-Rhinophoridae interaction

Very few studies regard the biology of the larva and its effect on the woodlouse host. These studies usually demand a long period of time due to the difficulty of obtaining the parasitoids (Thompson 1934, Bedding 1965, 1973). This difficulty is partially explained by the low prevalence of this parasitoid on natural populations and for the apparent specificity of host species (Bedding 1965). Prevalence on natural populations is usually lower than 2% and seems to be associated with the infection method.

Adult Rhinophoridae flies copulate and the female deposits the eggs on substrates (Bedding 1965, Wijnoven 2001) contaminated by uropod gland secretion of isopods rather than on the host itself (Bedding 1965) which may be a derived character in this group of parasitoids (Wood 1987). This secretion is not commonly observed in all woodlice species but it is rather easily obtained from *Porcellioscaber* Latreille, 1804 (Gorvett 1946, Deslippe et al. 1996) which might explain why this species has the highest number of known parasitoids and highest prevalence on natural populations (Bedding 1965, Sassaman and Pratt 1992).

The eggs deposited on the soil hatch and the 1^st^ instar larva attaches itself to the body of a passing woodlouse. The larva may wave its anterior end slowly forward and sideward in an attempt to attach itself to the body of a passing woodlouse (Pape and Arnaud 2001). This method of infection is affected by host size since the larva cannot reach the sternites of bigger (taller) animals. It was also observed the suitability of the host relates to a specific period of the molting cycle of the isopod. Differently from insects, crustaceans present a highly calcified cuticle (Roer et al. 2015). Within crustaceans, isopods have developed specific strategies to recycle calcium from the old cuticle such as a biphasic molting (they first molt the posterior half and then the anterior half of the body) and accumulation of amorphous calcium carbonate in the anterior sternites prior ecdysis (Greenaway 1985, Steel 1993, Ziegler 1994). The fly larva attaches itself to isopods with calcium plates (i.e., during premolt or intramolt) and penetrates through the intersegmental membrane of the sternites of the freshly molted host (Bedding 1965), since they present a softer cuticle at this stage. Nonetheless, there is a high rate of cannibalism of freshly molted isopods (Bedding 1965) thus reducing the chances of survival of the fly larva inside the host and possibly explaining the low prevalence among natural populations.

**Figure 1.**
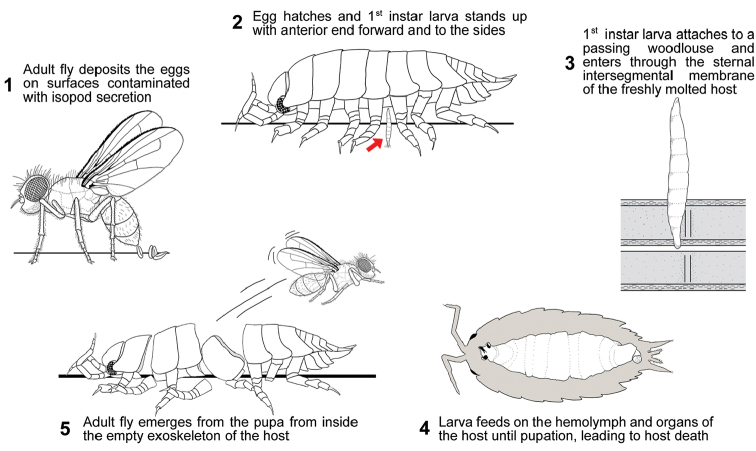
Schematic representation of the infection cycle of a Rhinophoridae fly in a woodlouse host. **3** is modified from Thompson (1934).

After the larva has entered the host, it then molts to its 2^nd^ instar and starts feeding, first on the hemolymph, and then on the organs of the host. The 3^rd^ instar larva fills most of the body cavity leading to isopod death. Pupation occurs inside the empty exoskeleton of the host (Thompson 1934, Bedding 1965) (Figure 1).

### First Neotropical woodlouse host record

Almost all records from Rhinophoridae hosts are from the Palearctic region. Outside the Palearctic, there is only mention of *Porcellioscaber*, *Oniscusasellus* Linnaeus, 1758 and *Porcellionidespruinosus* (Brandt, 1833) in the Nearctic (Brues 1903, Jones 1948, Sassaman and Garthwaite 1984, Sassaman and Pratt 1992) and *Armadillidium* sp. (probably *Armadillidiumvulgare* (Latreille, 1804)) in the Neotropic (Parker 1953). All of these woodlice species were parasitized by *Melanophoraroralis* (Linnaeus, 1758). Nonetheless, all the aforementioned oniscidean and rhinophorid species are introduced from the Palearctic on these locations. Some authors hypothesize that transportation of infected woodlice can explain the occurrence of Palearctic Rhinophoridae in the Nearctic and Neotropic (Mulieri et al. 2010, O’Hara et al. 2015) provided that introduced woodlice are common in these regions (Jass and Klausmeier 2000). The lack of native woodlouse hosts in the Nearctic region is thought to be associated with the low diversity of native woodlice species there (*c.f.*Schmalfuss 2003), but the same is not true for the Neotropic. In fact, in Brazil alone there is circa 200 described species, most of them native (Cardoso et al. 2018).

**Figure 2.**
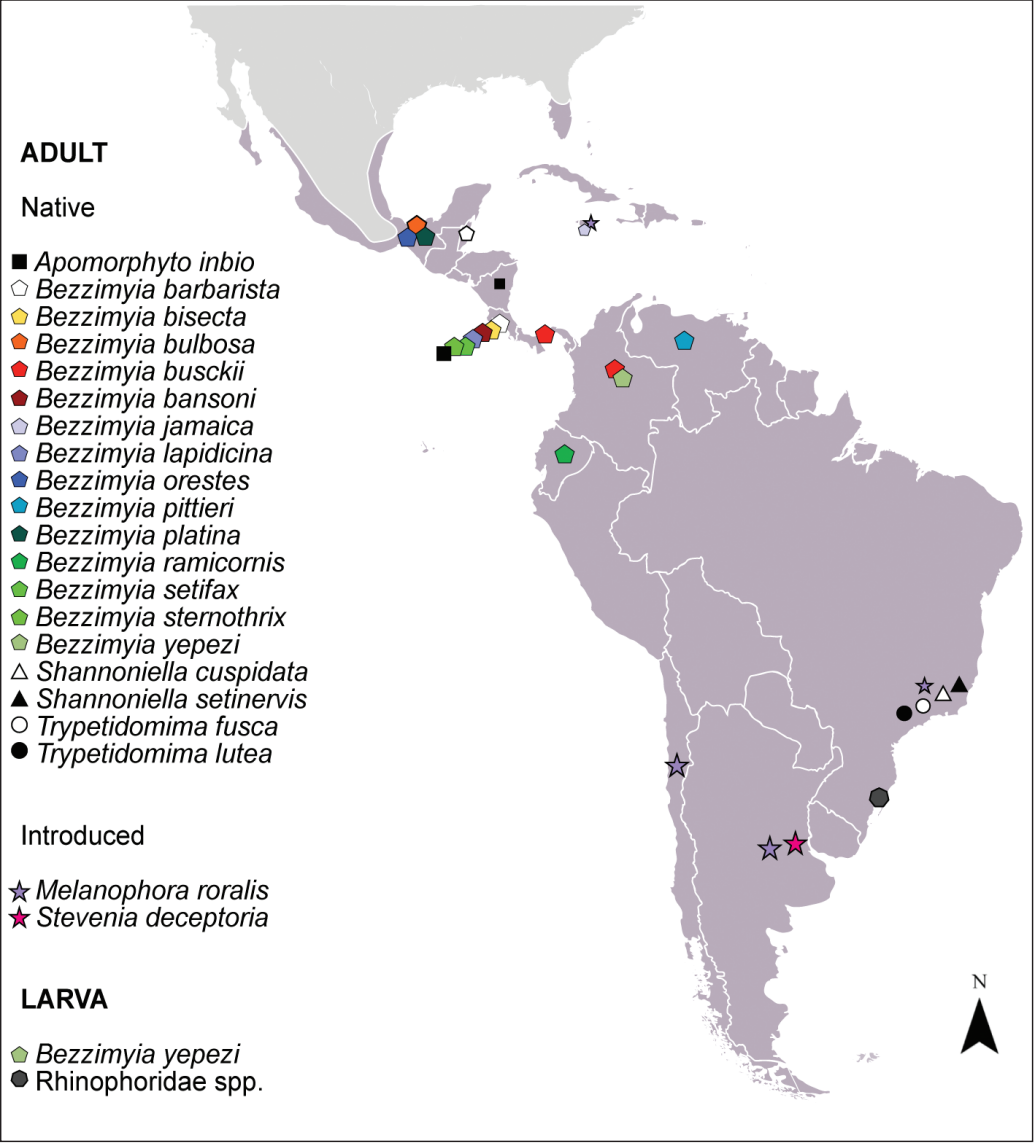
Distribution map of native and introduced Rhinophoridae species in the Neotropical region (area in gray) including the new larvae record. *Apomorphytoinbio* records from Pape (2010) and Cerretti et al. (2014); *Bezzimyia* spp. records from Pape and Arnaud (2001); *Shannoniella* spp. records from Nihei et al. (2016); *Trypetidomima* spp. records from Nihei and Andrade (2014); *Melanophoraroralis* records from Parker (1953), Guimarães (1977), González (1998), Cerretti and Pape (2009) and Mulieri et al. (2010); *Steveniadeceptoria* records from Mulieri et al. (2010). Base map modified from: commons.wikimedia.org/

In the Neotropic, 19 native species of Rhinophoridae have been described (Cerretti et al. 2014, Nihei et al. 2016), but there is no information regarding parasitoid-host interaction so far. Of these, only the 1^st^ instar larva of *Bezzimyiayepesi* Pape & Arnaud, 2001 (Venezuela) is known (Pape and Arnaud 2001) and no host record has been made before, even for the two introduced species, *Melanophoraroralis* (L.) and *Steveniadeceptoria* (Loew, 1847) (Mulieri et al. 2010) (Figure 2).

Here we observed that the Neotropical isopod *Balloniscusglaber* is a host for the dipterous larvae in southern Brazil (Figure 2), and the two observed 3^rd^ instar larvae morphotypes are different from the nine Palearctic species with previously described 3^rd^ instar larval forms (Thompson 1934, Bedding 1965), including the introduced *Melanophoraroralis*.

**Figure 3.**
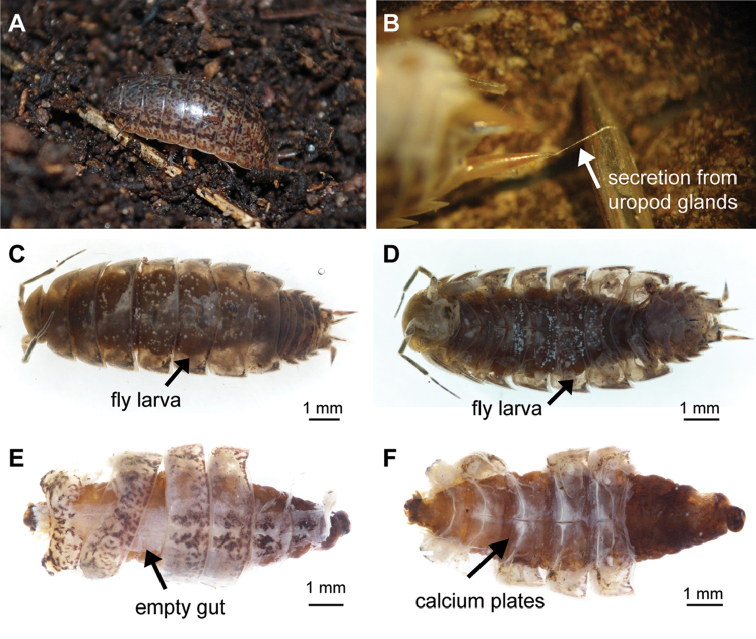
*Balloniscusglaber* infected with 3^rd^ instar Rhinophoridae larva **A** alive *B.glaber* clinging to the substrate **B** secretion discharged from uropod glands **C–D** dorsal (**C**) and ventral (**D**) views of *Balloniscusglaber* with a third instar Rhinophoridae larva inside **E–F** partially dissected *Balloniscusglaber* infected with Rhinophoridae larva showing the empty gut on dorsal view (**E**) and the calcium plates on ventral view (**F**).

**Figure 4.**
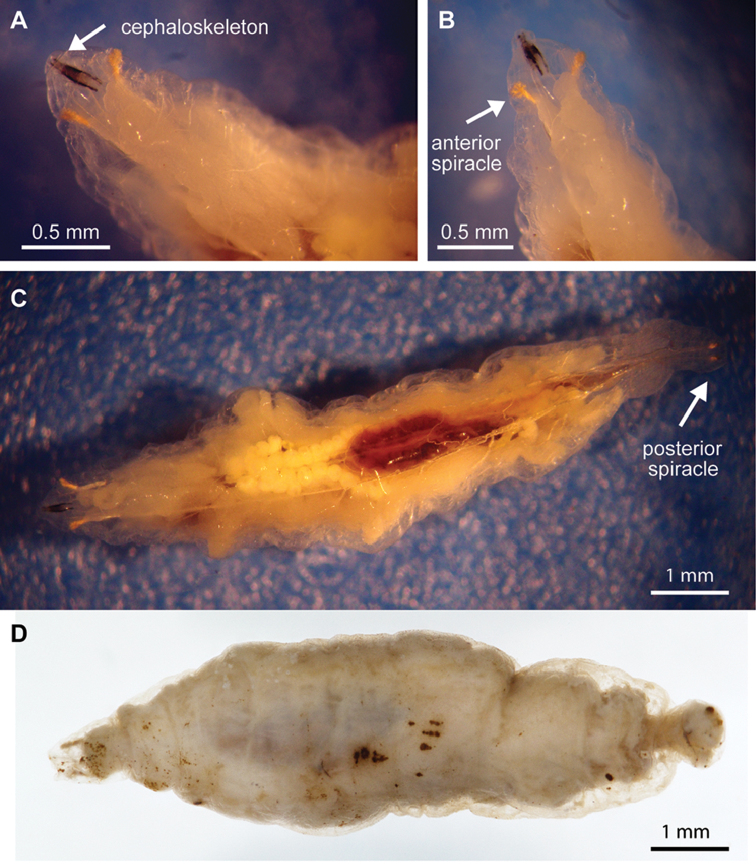
Rhinophoridae larva obtained from the Neotropical isopod *Balloniscusglaber*. Morphotype 1, 3^rd^ instar **A** detail view of the cephaloskeleton on dorsal view **B** detail view of the anterior spiracle **C** alive larva with transparent integument **D** fixed larva, dorsal view.

**Figure 5.**
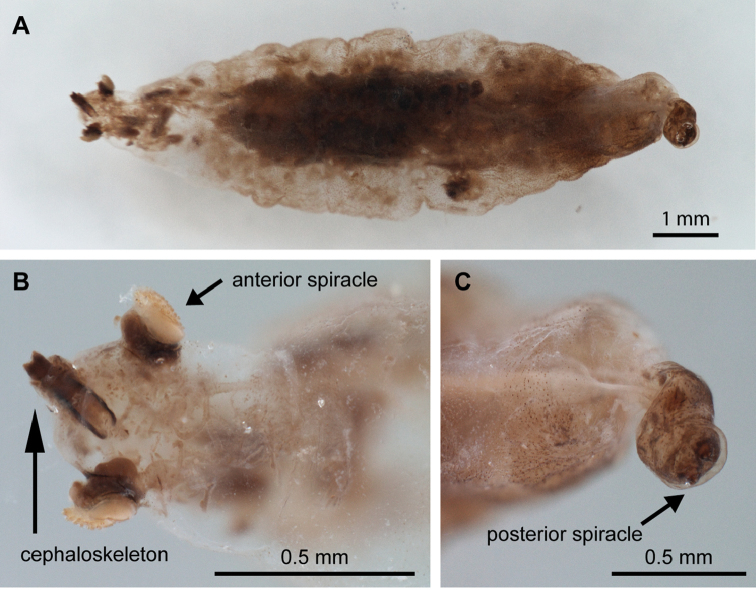
Rhinophoridae larva obtained from the Neotropical isopod *Balloniscusglaber*. Morphotype 2, 3^rd^ instar **A** dorsal view **B** detail from anterior part showing the cephaloskeleton and anterior spiracles **C** detail of the posterior spiracle.

*Balloniscusglaber* shares many characteristics with clingers (Wood et al. 2017) although it does not present a typical clinger eco-morphological body type like *Porcellioscaber* (*sensu*Schmalfuss 1984). However, it presents clinging behavior (Figure 3A) for predator avoidance (Quadros et al. 2012, Wood et al. 2017) and its legs are shorter than in runner type animals of similar size. These morphological and behavioral characteristics might facilitate larva infection due to reduced distance of sternites to the substrate. Furthermore, like *Porcellioscaber*, this species also frequently discharges a sticky secretion from their uropod glands upon stimulation (Figure 3B), secretion that is recognized by adult fly females and might stimulate oviposition (Bedding 1965). Five infected individuals have been recorded in the same location (Figure 3C–F). The larvae (one per host) occupied the full body cavity, reaching up to 7 mm in length and resulted in death of all woodlice hosts (Suppl. material 2). Hosts lacked discernible internal reproductive system and the empty gut was the only remaining organ (Figure 3E). No host presented any signs of alteration in overall appearance. The parasitoids could only be identified at the family level due to the lack of larval descriptions for the native species and lack of adults to get a more precise identification. The larvae were identified as Rhinophoridae based on comparative examination of descriptions and illustrations available on the literature; both collected morphotypes presented elongate body shape, anterior and posterior spiracles, and cephaloskeleton as characterized by rhinophorid species. The two 3^rd^ instar larvae morphotypes are conspicuously different on body shapes, posterior ends, cephaloskeleton, and anterior and posterior spiracles (Figs 4, 5). These forms differ from the known larval stages described by Thompson (1934) and Bedding (1965, 1973). Given the apparent specificity of host records (see next topic) we believe they are Neotropical species (and none of the introduced species). They may be larvae of the described Neotropical species of *Shannoniella* Townsend, 1939 or *Trypetidomima* Townsend, 1935, or they may even belong to undescribed species, since the distribution of *Balloniscusglaber* (Lopes et al. 2005) does not extend to the locations where these native Rhinophoridae have been found, namely, the southeastern portion of Brazilian Atlantic Forest (Nihei and Andrade 2014, Nihei et al. 2016). Furthermore, the location of the new Rhinophoridae record is at a low altitude and Neotropical woodlouse flies seem to be rare in the lowlands, being usually found at elevations of 600–1200 meters in Brazil (Nihei and Andrade 2014, Nihei et al. 2016). Nonetheless, *Balloniscusglaber* can be found in altitudes up to 1000 meters in southern latitudes (Lopes et al. 2005) while another species from the genus, *Balloniscussellowii* (Brandt, 1833), presents a broader latitudinal distribution (Schmalfuss 2003).

A further publication will describe in detail the morphology of the two 3^rd^ instar morphotypes, and DNA sequencing will be performed trying to obtain a more precise identification.

### Reviewed interactions records following current taxonomy

The earliest reference to a Rhinophoridae parasitoid of woodlice appears to be from von Roser (1840 *apud*Thompson 1934) that created some confusion in the literature in later years. In his paper, the dipteran “*Tachiniaatramentaria*” (currently *Steveniaatramentaria* (Meigen, 1824)) is mentioned as a parasite of a woodlouse, possibly *Oniscusasellus*. Thompson (1934), Herting (1961), Bedding (1965) and Verves and Khrokalo (2010) mentioned that *Oniscusasellus* was probably a wrong identification while Cerretti and Pape (2007) mention *Oniscusasellus* as a possible host for *Steveniaatramentaria*. The doubtful record was finally resolved in Kugler (1978) where the author states the record was based on a misidentification of *Trachelipusrathkii* (Brandt, 1833) according to a personal communication from Herting, that apparently had already been corrected in Sutton’s book (1980). Rognes (1986) and Dubiel and Bystrowski (2016) still list *Oniscusasellus* as a host or possible host of *Steveniaatramentaria* but referencing articles that mention the species as a possible host, probably following von Roser’s reference from 1840. Therefore, we could not find any reliable record of *Oniscusasellus* as a host of *Steveniaatramentaria*. Dubiel and Bystrowski (2016) list *Trachelipusrathkii* as a host from *Steveniaatramentaria* for the first time, but it should be the third record of this interaction if the identification correction from von Roser’s article is taken into account as well as the thesis from Bedding (1965).

The following record on the literature is from Brues (1903) indicating *Melanophoraroralis* as a parasitoid of *Porcellio* sp., probably *Porcellioscaber* in Massachusetts, USA. Besides *Porcellioscaber* as a host, this dipteran species was also recorded as a parasitoid of *Oniscusasellus* (Jones 1948, Sassaman and Garthwaite 1984, Sassaman and Pratt 1992) and *Porcellionidespruinosus* (Sassaman and Garthwaite 1984) in the United States. In the Palearctic region, besides the aforementioned isopods, *Porcelliospinicornis* Say, 1818 (Irwin 1983) is also listed as host for *Melanophoraroralis*. This species of fly shows the highest plasticity of hosts as well as largest geographical distribution that is not restricted to the Palearctic region. It is found in the U.S.A. (Brues 1903, Jones 1948, Sassaman and Garthwaite 1984, Cerretti and Pape 2009), Chile (González 1998), Argentina, Brazil and Jamaica (Guimarães 1977, Mulieri et al. 2010).

Records of interactions are available in von Roser (1840 *apud*Thompson 1934), Brues (1903), Thompson (1934), Jones (1948), Parker (1953)Herting (1961)Bedding (1965), Kugler (1978), Irwin (1983), Sassaman and Garthwaite (1984), Nash (1985), Bürgis (1991, 1992), Cordaux et al. (2001), Wijnhoven (2001), Cerretti et al. (2014) and Dubiel and Bystrowski (2016) although most of this literature is not taxonomically updated. Other works like Arnaud (1978), Draber-Monko (1981), Rognes (1986), Wijnhoven and Zeegers (1999), Cerretti and Pape (2007), and Ziegler (2008) report known hosts from the literature (some of them with current species’ names), but don’t present new records. Therefore, the number of interactions is certainly higher than it has been recorded so far. Currently, there are 18 Isopoda species known to be parasitized (one with an undetermined Rhinophoridae species), and 13 Rhinophoridae species with known hosts, resulting in 35 known interactions (and two others lacking host species identification) and a total of 53 records from 12 countries. Out of the 18 known isopod hosts, only five species have more than one parasitoid: *Porcellioscaber* (seven or eight rhinophorid species), *Oniscusasellus* (four spp.), *Trachelipusrathkii* (three spp.), *Armadillidiumvulgare* (two or three spp.), *Porcelliospinicornis* and *Porcellionidespruinosus* (two spp.), and *Balloniscusglaber* (with two undetermined morphotypes recorded here) (Table 1).

**Table 1.** Records of Isopoda-Rhinophoridae (host-parasitoid) interactions from the literature with updated taxonomy. The records from Bedding 1965 are also presented in Sutton (1980). Supplementary dataset presents, additionally, the name of the species of both Rhinophoridae and Isopoda in the original record publication, country, and biogeographical region Suppl. material 1.

Isopod species	Rhinophoridae species	Source
**Family Armadillidae**		
* Armadillidium frontirostre *	* Stevenia signata *	Bürgis 1992
* Armadillidium nasatum *	* Phyto melanocephala *	Herting 1961 (after Legrand)
	Rhinophoridae sp.	Cordaux et al. 2001
* Armadillidium silvestrii *	* Phyto melanocephala *	Herting 1961 (Verhoeff after Séguy 1941)
* Armadillidium versicolor *	* Phyto melanocephala *	Herting 1961 (Verhoeff after Séguy 1941)
* Armadillidium vulgare *	* Phyto melanocephala *	Thompson 1934 (found by Donisthorpe); Bedding 1965
	* Stevenia signata *	Bürgis 1991, 1992
*Armadillidium* sp. (probably *A.vulgare*)	* Melanophora roralis *	Parker 1953
* Armadillo officinalis *	* Phyto armadillonis *	Kugler 1978
**Family Balloniscidae**		
* Balloniscus glaber *	Rhinophoridae sp.	Wood et al. 2018 (present study)
**Family Cylisticidae**		
* Cylisticus convexus *	* Rhinomorinia sarcophagina *	Dubiel and Bystrowski 2016
**Family Oniscidae**		
* Oniscus asellus *	* Melanophora roralis *	Jones 1948
	* Paykullia maculata *	Thompson 1934, Bedding 1965
	* Phyto discrepans *	Thompson 1934, Bedding 1965
	* Phyto melanocephala *	Thompson 1934 (found by Donisthorpe)
**Family Philosciidae**		
* Philoscia affinis *	* Stevenia atramentaria *	Herting 1961 (Verhoeff after Séguy 1941)
**Family Porcellionidae**		
* Porcellio laevis *	* Phyto luteisquama *	Kugler 1978
* Porcellio scaber *	* Melanophora roralis *	Thompson 1934, Jones 1948, Bedding 1965, Sassaman and Garthwaite 1984
	* Paykullia maculata *	Thompson 1934, Bedding 1965
	* Phyto discrepans *	Thompson 1934, Bedding 1965
	* Phyto melanocephala *	Thompson 1934, Bedding 1965
	* Rhinophora lepida *	Thompson 1934, Bedding 1965
	* Stevenia atramentaria *	Thompson 1934
	* Tricogena rubricosa *	Thompson 1934, Bedding 1965, Nash 1985, Dubiel and Bystrowski 2016
	*Stevenia* sp.	Cordaux et al. 2001
*Porcellio* sp. (probably *Porcellioscaber*)	* Melanophora roralis *	Brues 1903
	* Stevenia deceptoria *	Cerretti et al. 2014
* Porcellio spinicornis *	* Melanophora roralis *	Irwin 1983
	* Phyto melanocephala *	Herting 1961 (Legrand after Seguy 1941)
* Porcellionides pruinosus *	* Melanophora roralis *	Sassaman and Garthwaite 1984
	* Phyto angustifrons *	Thompson 1934
* Protracheoniscus politus *	* Paykullia maculata *	Herting 1961 (Verhoeff after Séguy 1941)
**Family Trachelopodidae**		
* Trachelipus arcuatus *	* Stevenia atramentaria *	Herting 1961 (Verhoeff after Séguy 1941)
* Trachelipus rathkii *	* Paykullia maculata *	Wijnhoven 2001
	* Stevenia atramentaria *	von Roser 1840 (corrected in Kugler 1978 by Herting as personal communication), Bedding 1965, Dubiel and Bystrowski 2016
	* Tricogena rubricosa *	Dubiel and Bystrowski 2016
* Trachelipus ratzeburgii *	* Paykullia maculata *	Herting 1961

## Supplementary materials

Supplementary material 1 Compilation of Isopoda-Rhinophoridae records (Dataset) Data type: species data This is an open access article distributed under the terms of the Creative Commons Attribution License (CC BY 4.0), which permits unrestricted use, distribution, and reproduction in any medium, provided the original author and source are credited.Camila T Wood, Silvio S Nihei, Paula B Araujo

Supplementary material 2 Rhinophoridae larva from a Neotropical woodlouse species Data type: multimedia This is an open access article distributed under the terms of the Creative Commons Attribution License (CC BY 4.0), which permits unrestricted use, distribution, and reproduction in any medium, provided the original author and source are credited.Camila T Wood, Silvio S Nihei, Paula B Araujo
